# American Dental Association

**Published:** 1867-07

**Authors:** J. Taft, W. H. Goddard, H. R. Smith

**Affiliations:** Committee of Arrangements.; Committee of Arrangements.; Committee of Arrangements.


### *American Dental Association.*

THIS body meets, according to adjournment, in the city of Cincinnati, on the last Tuesday of July. It is confidently expected that there will be a large attendance, probably larger than ever before. In order that the meeting be one of interest and profit, all the members should come with the full purpose of accomplishing the most possible. Every one should come with a purpose and a preparation to do his full share of the work. The responsibility of making the meeting an interesting one does not rest upon the officers, nor upon any committee, but it does rest upon *all the members* who may be present, and hence we hope that every one will bring his contribution, and time shall be afforded for its presentation.

The time of the Association, so far at least as the arrangements of the Executive Committee are concerned, shall be devoted exclusively to its legitimate work; outside attractions will be wholly ignored, during the time of the sessions. We say this because of a thorough conviction that the members of the Association wish to have it so.

The Executive Committee have made ample arrangements for the accommodation of the Association; Hopkins' Music Hall, on 4th street, near Elm, a very excellent room, has been secured. There are sufficient ante rooms, and a fine room for clinics.

The Committee will be at the room as early as 8 o'clock on the morning of the first day, for the purpose of receiving the credentials of the new members. It is desirable that that part of the business be done before the regular hour of meeting, so far as practicable; Delegates will, therefore, report early.

Arrangements have been made for the accommodation of the members, with the Burnet House, Clarendon Hotel, and the St. James, in either of which the accommodation and arrangements will be all that the most fastidious could desire. Any parties wishing to secure rooms, can do so by notifying Dr. H. R. Smith, of Cincinnati, who will give prompt attention to such requests.

Very efficient arrangements have been made for the presentation and exhibition of instruments and appliances. We hope that all members having anything new and valuable, and even though it may be old and valuable, and not generally known to the profession, will not fail to bring it in. A fixed time each day has been assigned for each exhibition.

Any articles for exhibition, consigned to Dr. H. R. Smith, will be received and taken care of by him.

J. Taft,

W. H. Goddard,

H. R. Smith,

*Committee of Arrangements.*

